# Tumor necrosis targeted radiotherapy of non-small cell lung cancer using radioiodinated protohypericin in a mouse model

**DOI:** 10.18632/oncotarget.4568

**Published:** 2015-07-21

**Authors:** Xuejiao Liu, Cuihua Jiang, Dongjian Zhang, Meng Gao, Fei Peng, Dejian Huang, Ziping Sun, Yicheng Ni, Jian Zhang, Zhiqi Yin

**Affiliations:** ^1^ Laboratory of Translational Medicine, Jiangsu Province Academy of Traditional Chinese Medicine, Nanjing 210028, Jiangsu Province, P.R.China; ^2^ College of Pharmacy, Nanjing University of Chinese Medicine, Nanjing 210023, Jiangsu Province, P.R.China; ^3^ Shandong Academy of Medical Sciences, Jinan 250062, Shandong, P.R.China; ^4^ Theragnostic Laboratory, Campus Gasthuisberg, KU Leuven, 3000 Leuven, Belgium; ^5^ Department of Natural Medicinal Chemistry & State Key Laboratory of Natural Medicines, China Pharmaceutical University, Nanjing 210009, Jiangsu Province, P.R.China

**Keywords:** tumor necrosis targeted radiotherapy, necrosis-avid agent, radiopharmaceutical, protohypericin, drug combination

## Abstract

Lung cancer is the leading cause of cancer-related death. About 80% of lung cancers are non–small cell lung cancers (NSCLC). Radiotherapy is widely used in treatment of NSCLC. However, the outcome of NSCLC remains unsatisfactory. In this study, a vascular disrupting agent (VDA) combretastatin-A4-phosphate (CA4P) was used to provide massive necrosis targets. ^131^I labeled necrosis-avid agent protohypericin (^131^I-prohy) was explored for therapy of NSCLC using tumor necrosis targeted radiotherapy (TNTR). Gamma counting, autoradiography, fluorescence microscopy and histopathology were used for biodistribution analysis. Magnetic resonance imaging (MRI) was used to monitor tumor volume, ratios of necrosis and tumor doubling time (DT). The biodistribution data revealed ^131^I-prohy was delivered efficiently to tumors. Tracer uptake peaked at 24 h in necrotic tumor of ^131^I-prohy with and without combined CA4P (3.87 ± 0.38 and 2.96 ± 0.34%ID/g). ^131^I-prohy + CA4P enhanced the uptake of ^131^I-prohy in necrotic tumor compared to ^131^I-prohy alone. The TNTR combined with CA4P prolonged survival of tumor bearing mice relative to vehicle control group, CA4P control group and ^131^I-prohy control group with median survival of 35, 20, 22 and 27 days respectively. In conclusion, TNTR appeared to be effective for the treatment of NSCLC.

## INTRODUCTION

Lung cancer is the leading cause of cancer-related death worldwide. Non–small cell lung cancer (NSCLC) accounts for about 85% of all cases of lung cancer [1]. Patients with NSCLC have distressingly short survival times following diagnosis. Radical surgical resection offers potential cure of NSCLC. One third of patients with early stage of NSCLC are treated with surgery [2]. However, medically inoperable patients will be considered for radiotherapy [3, 4].

Radiotherapy is widely used in both curative and palliative treatment of NSCLC. With the development of radiotherapy techniques, three-dimensional conformal radiation therapy showed better local control and possibly better survival rates than traditional two-dimensional radiation therapy for patients with medically inoperable stage I NSCLC [5]. Precise definition of the tumor's anatomical extent is fateful for accurate location and shaping of the radiotherapy beams. Image-guided techniques such as ^18^F-FDG PET/CT scanning can improve the targeting accuracy of NSCLC [6]. Due to respiration and cardiac motion, tumor motion during radiation treatment must be considered and addressed. Four-dimensional CT scanning enables accurate individualize localization and radiotherapy based on tumor motion [7]. These advanced radiotherapy techniques greatly improved the treatment of NSCLC. However, two obstacles still exist in the control and eradication of NSCLC: 1) these techniques are suitable for locoregional disease rather than systemic therapy, for patients with metastatic disease, the curative effect of these methods is unlikely; and 2) biological effective doses to eradicate tumor cannot be delivered safely due to the risk of normal tissue toxicity.

Tumor targeted radiotherapy provides a challenging approach for cancer therapy to overcome these obstacles. Tumor necrosis treatment (TNT) is one of tumor targeted radiotherapy method based on the hypothesis that monoclonal antibodies which target abundant and universal intracellular antigens retained by dying cells will preferentially localize to necrotic regions of tumors [8]. Radionuclide combined with monoclonal antibodies can be delivered to necrotic regions of tumors and emits radiation to kill and/or restrain adjacent cancer cells. Iodine-131 radiolabeled chimeric tumor necrosis therapy monoclonal antibody (^131^I-chTNT) has been approved for the treatment of advanced lung cancer in China. A study showed ^131^I-chTNT was well tolerated with excellent localization in tumors and 33% objective response rate in NSCLC patients [9]. However, the macromolecule immunogenicity of monoclonal antibodies and the adverse side effect bone marrow suppression became the main reasons for limiting its application [10].

A substitute approach using small necrosis targeted molecule can solve the problem of immunogenicity. Recent years have witnessed accelerated progress in preclinical development of necrosis targeted agents that potentially could be used for TNTR. This method will provide a novel approach for the treatment of NSCLC. Protohypericin (prohy) is a polycyclic aromatic dianthraquinone derived from plant *Hypericum perforatum*, and it can be synthesized using emodin as the starting molecule. In our previous studies, ^131^I-prohy was discovered with a unique affinity to necrotic tissues in rat model of reperfused hepatic infarction with a plasma elimination half-life of 14.9 h [11]. Whether radionuclide can be delivered with prohy efficiently to tumors or not was investigate in this study by detecting the biodistribution, autoradiography and histopathology.

In addition, Combretastatin-A4-phosphate (CA4P) is a tumor vascular disrupting agent (VDA) that can cause rapid, selective, and extensive vascular damage, resulting in extensive ischemic necrosis [12]. These necrotic regions provide perfect targets for necrosis-avid agents. Therefore, radionuclide labeled necrosis-avid agents combined with a VDA may produce a synergetic method for TNTR. To validate this approach for the treatment of NSCLC, we investigated the anticancer efficacy of ^131^I-prohy in combination with CA4P in a human NSCLC murine xenotransplant model by analyzing the survival probability and magnetic resonance imaging.

## RESULTS

### Animals and tumor model

Unilateral A549 tumor-bearing nude mice models were established successfully. All mice survived the surgery and anesthesia procedures without any drug administration related deaths.

### Biodistribution analysis

Data were exhibited in Table 1. The highest radioactivity retention of ^131^I-prohy group and CA4P + ^131^I-prohygroup occurred in almost all normal tissues at early time point (4 h) after administration, and decreased rapidly thereafter up to 120 h except necrotic tumor. Relatively high uptakes of ^131^I-prohy were found in the liver, viable tumor and necrotic tumor through the detecting time points. The ^131^I-prohy radioactivity accumulation of CA4P + ^131^I-prohy group in necrotic tumor was significantly higher than that of ^131^I-prohy group at 4 h, 24 h and 120 h (*p* < 0.05). The radioactivity quantification of dissected tumor showed higher uptake in necrotic tumor than viable tumor. The necrosis-to-viable tumor ratios were 0.9, 2.3, 2.8 in ^131^I-prohy group, and 1.0, 2.6, 5.3 in CA4P + ^131^I-prohy group at 4 h, 24 h and 120 h.

**Table 1:** Biodistribution of ^131^I-prohy and CA4P + ^131^I-prohy in A549 tumor bearing nude mice at 4 h, 24 h and 120 h post injection (*n* = 6 per time point)

Organs	%ID/g					
	4 h		24 h		120 h	
	^131^I-prohy	CA4P + ^131^I-prohy	^131^I-prohy	CA4P + ^131^I-prohy	^131^I-prohy	CA4P + ^131^I-prohy
Blood	4.43 ± 0.14	4.75 ± 0.65	0.66 ± 0.08	0.74 ± 0.03	0.06 ± 0.01	0.09 ± 0.02*
Thyroid	0.47 ± 0.03	1.14 ± 0.07**	0.08 ± 0.02	0.36 ± 0.02**	0.12 ± 0.03	0.25 ± 0.11
Lung	2.71 ± 0.23	2.64 ± 0.23	1.68 ± 0.16	1.27 ± 0.40	0.20 ± 0.04	0.26 ± 0.08
Heart	1.26 ± 0.11	1.61 ± 0.13*	0.54 ± 0.16	0.32 ± 0.02	0.24 ± 0.07	0.12 ± 0.02*
liver	5.60 ± 0.38	5.84 ± 0.40	2.13 ± 0.38	1.88 ± 0.24	0.70 ± 0.10	0.75 ± 0.08
Spleen	2.48 ± 0.06	2.98 ± 0.46	1.05 ± 0.08	0.74 ± 0.10*	0.31 ± 0.01	0.38 ± 0.05
Kidney	2.61 ± 0.29	2.75 ± 0.09	1.22 ± 0.13	1.24 ± 0.19	0.13 ± 0.01	0.19 ± 0.02*
Stomach	0.74 ± 0.14	1.14 ± 0.27	0.44 ± 0.08	0.47 ± 0.09	0.07 ± 0.01	0.14 ± 0.02*
Muscle	0.45 ± 0.15	0.38 ± 0.01	0.10 ± 0.02	0.15 ± 0.05	0.02 ± 0.00	0.06 ± 0.01**
Bone	1.57 ± 0.11	1.34 ± 0.11	0.26 ± 0.06	0.40 ± 0.14	0.10 ± 0.01	0.09 ± 0.01
Small intestine	2.36 ± 0.24	2.20 ± 0.40	0.28 ± 0.15	0.33 ± 0.03	0.09 ± 0.01	0.11 ± 0.03
Larger intestine	1.23 ± 0.09	1.70 ± 0.31	0.33 ± 0.12	0.54 ± 0.08	0.06 ± 0.01	0.15 ± 0.05*
Necrotic Tumor	1.99 ± 0.29	3.01 ± 0.35*	2.96 ± 0.34	3.87 ± 0.38*	1.45 ± 0.31	2.14 ± 0.19*
Viable Tumor	2.16 ± 0.71	3.05 ± 0.38	1.26 ± 0.31	1.48 ± 0.04	0.51 ± 0.02	0.41 ± 0.09

Data are expressed as percentage injected dose per gram of tissue (%ID/g).

* *P* < 0.05

** *P* < 0.01 (comparison between ^131^I-prohy group and CA4P + ^131^I-prohy group)

### Autoradiography and histopathology

**Figure 1:**
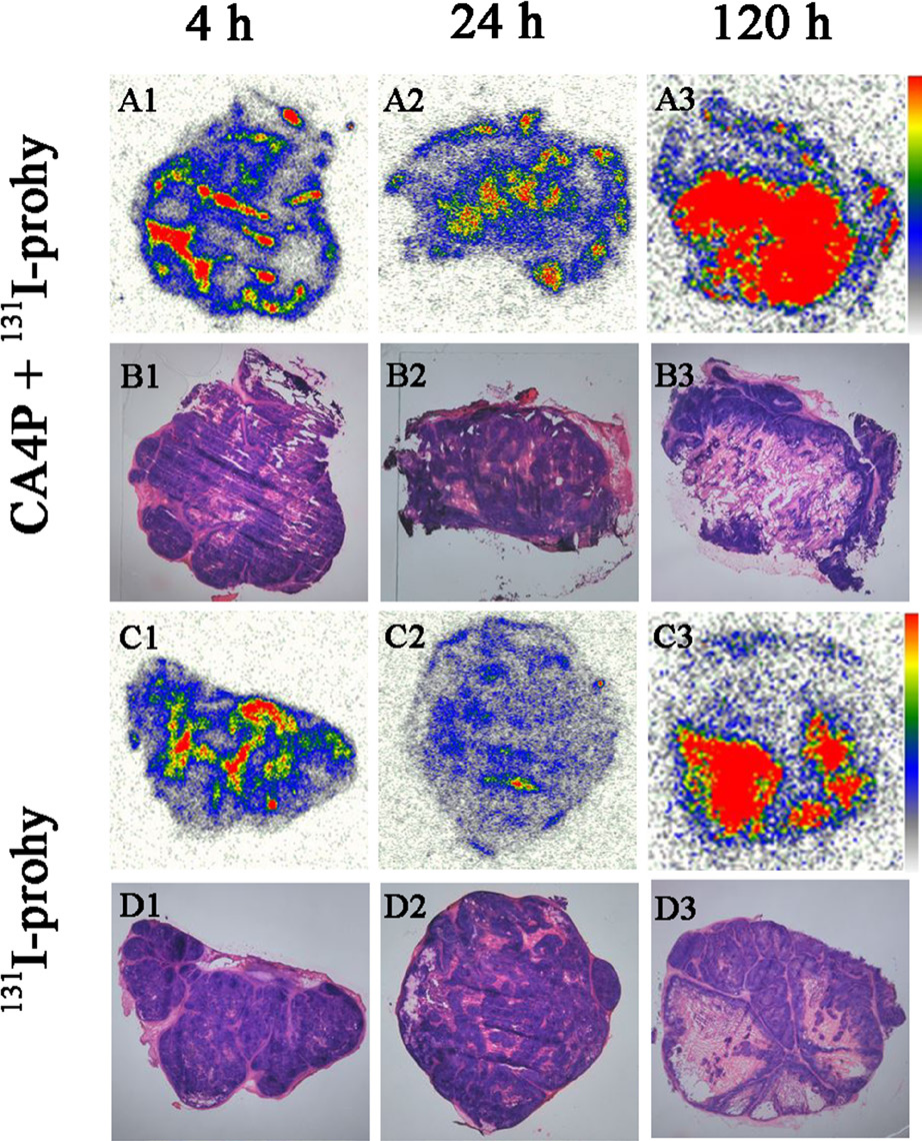
Autoradiograph (A1–A3, C1–C3) and corresponding H&E stained images (B1–B3, D1–D3) of CA4P + ^131^I-prohy group and ^131^I-prohy group at 4 h (A1, B1, C1, D1), 24 h (A2, B2, C2, D2) and 120 h (A3, B3, C3, D3)

Figure 1 represents typical images of intratumoral biodistribution of ^131^I-prohy for CA4P + ^131^I-prohy group and ^131^I-prohy group at 4 h, 24 h and 120 h. At each time point, higher tracer uptake appeared mainly in the necrotic tumor regions, which could be distinguished basing on the histopathological examination with H&E staining (The dark purple regions were viable tumor and the light pink parts were necrotic tumor). These results were consistent with the biodistribution data. The necrosis-to-viable tumor ratios detected by a semiquantitative autoradiography were 1.3, 9.3, 5.9 in ^131^I-prohy group, and 2.6, 10.3, 15.5 in CA4P + ^131^I-prohy group at 4 h, 24 h and 120 h.

### Intratumoral location of prohy

**Figure 2:**
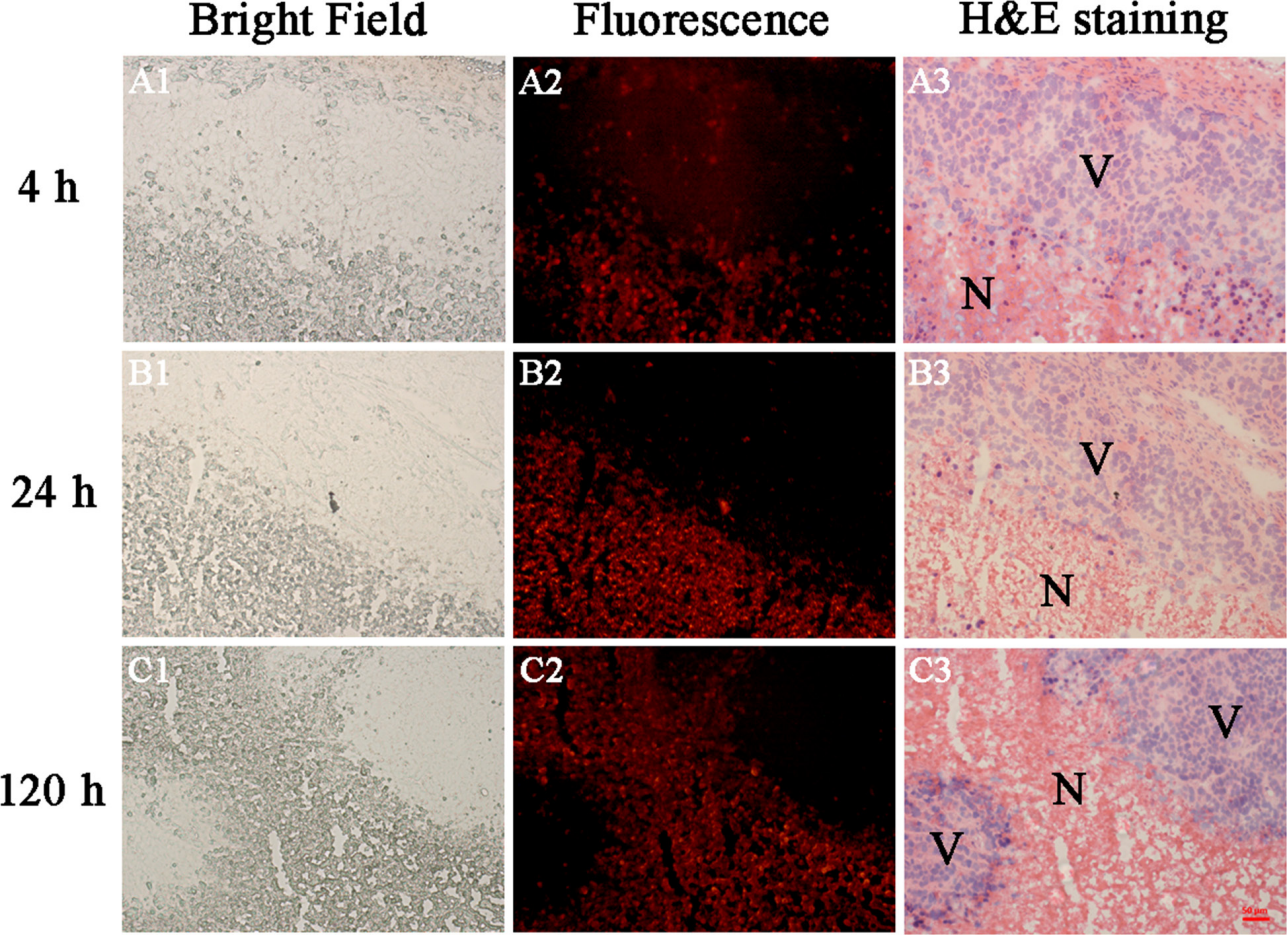
Images of 5 μm frozen tumor sections from A549 tumor bearing nude mice at 4 h, 24 h and 120 h after administration Corresponding bright field **(A1, B1, C1)**, fluorescence **(A2, B2, C2)** and H&E stained images **(A3, B3, C3)**. Prohy emitted red fluorescence. N=necrotic area, V=viable tumor. Scale bar = 50 μm.

In order to visualize the selective accumulation of prohy at an accurate microscopic level, intratumoral biodistribution was analyzed by fluorescence microscopy. Based on the red fluorescence of prohy and H&E staining, higher prohy uptake appeared in the necrotic tumor regions (Figure 2) with the necrotic/viable tumor ratios of 1.1, 28.8, and 41.7 at 4 h, 24 h, and 120 h, respectively. The accumulation of prohy at 4 h showed comparative fluorescence intensity in necrotic tumor and viable tumor, and this ratio increased at 24 h and 120 h. This tendency was consistent with gamma counting and autoradiography. In view of the gamma counting, autoradiography and fluorescence intensity data, it can be concluded that prohy first accumulated at both necrotic tumor and viable tumor at early time point (4 h), and mainly located in necrotic tumor after 24 h.

### Tumor necrosis targeted radiotherapy study

#### Magnetic resonance imaging (MRI)

**Figure 3:**
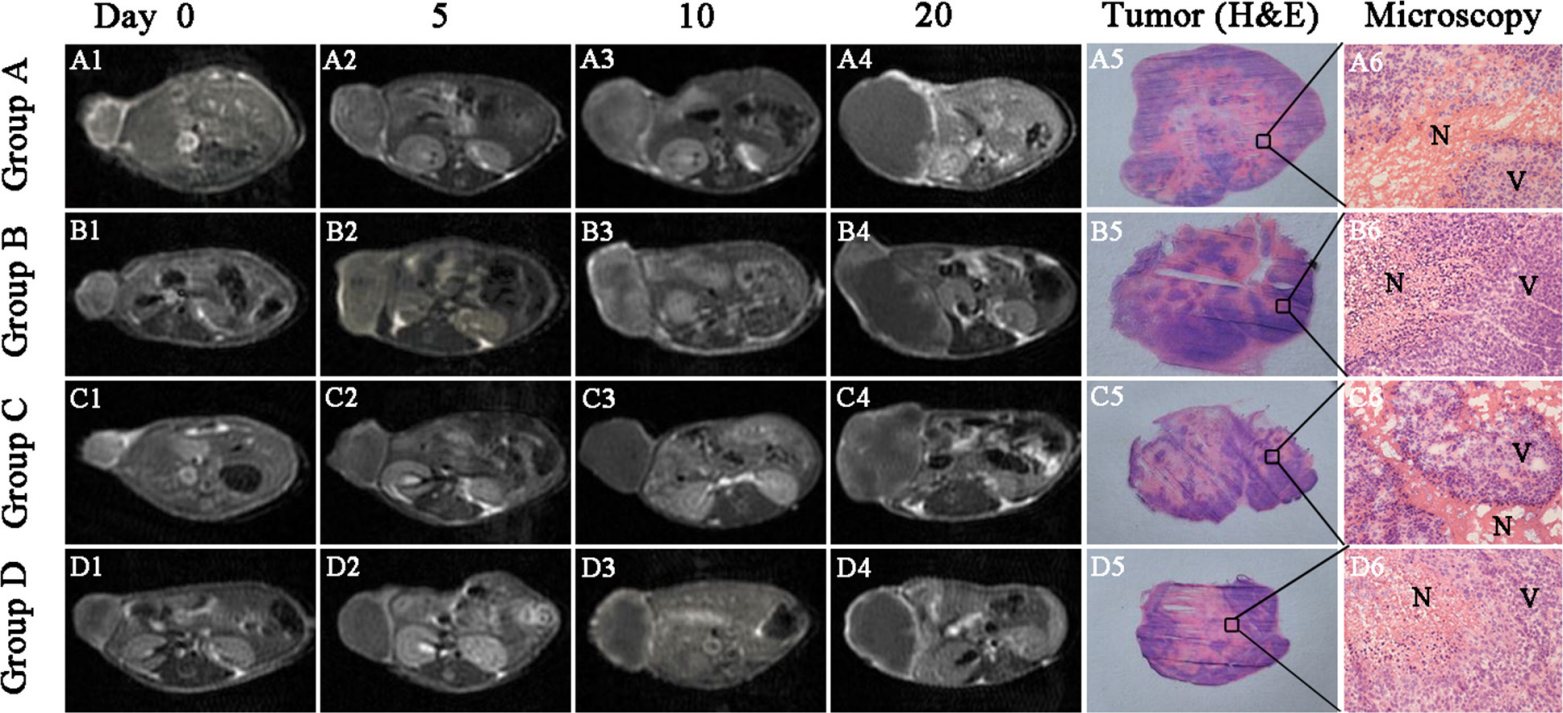
Contrast enhanced T1 (CE-T1) MR images of tumor bearing nude mice from 4 groups at day 0, 5, 10, 20 Tumors in group **A. B.** grew much faster than that of group **C.** and **D.** Macroscopic tumor (H&E) photographs (A5–E5) showed extensive central necrosis surrounded by some viable tumor tissues in all groups of nude mice at endpoint. Microscopic photographs (A6–E6) showed the interface between necrotic (N) and viable (V) tumor tissues in the 4 groups. Scale bar = 50 μm.

Tumors at baseline (Day 0) appeared slightly hypointense or isointense on T1WI and hyperintense on T2WI. On CE-T1 images, A549 tumors were enhanced after administration of Magnevist, suggesting the hypervascularity of the tumor. The groups administrated with CA4P (group B and D) showed a non-enhanced central region surrounded by a thin rim enhancement on CE-T1 images, indicating the presence of massive necrosis and minimum viable tumor region (Figure 3). However, the groups without CA4P (group A and C) also showed some necrotic region due to the spontaneous necrosis caused by a proportion of degenerating or dead cells in addition to numerous proliferating cells in rapidly growing tumors [8]. Necrosis and viable tumor could be distinguished by H&E stained microscopy (Figure 3 A6–E6). Derived from the images, tumors in group A, B grew much faster than that of group C and D.

### Survival analysis

**Figure 4:**
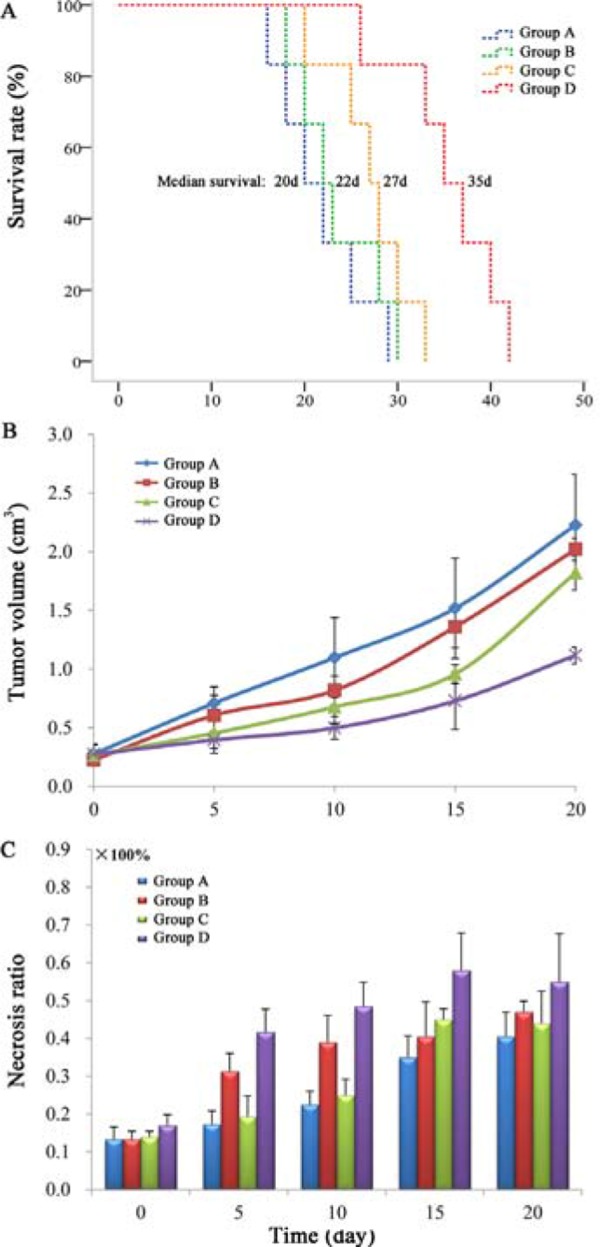
A. Kaplan-Meier survival curves show the survival rate (%) of the 4 groups of nude mice. The median survival was 20 (range 16–29), 22 (range 18–30), 27 (range 20–33) and 35 (range 26–42) days in group A, B, C and D respectively Tumor growth curve **B.** and corresponding necrosis ratios **C.** measured from MRI at baseline (day 0), and at day 5, 10, 15 and 20 after treatment. Significant difference of tumor volume in group **D** was found compared with that of group A, B and C (P < 0.05) from day 10 on. A significantly increased necrosis ratio (*p* < 0.05) was obtained in group B and D after CA4P injection.

The groups administrated radiopharmaceutical (Group C and Group D) prolonged survival of tumor bearing nude mice, the median survival in group A, B, C and D was 20 (range 16–29), 22 (range 18–30), 27 (range 20–33) and 35 (range 26–42) days respectively (Figure 4A). Group D showed significantly longer survival compared with group A, B (*p* < 0.01) and group C (*p* < 0.05). No significant difference was found among group A, B and C, but group C showed longer average survival days than group A and B.

### Tumor volume, tumor doubling time and necrosis ratio

Tumor volumes (Figure 4B) and the corresponding necrosis ratio (Figure 4C) were calculated by MRI on day 0, 5, 10, 15, 20. Tumor volumes at baseline (Day 0) were approximate in group A, B, C and D, respectively (*p* > 0.05). Significant difference of tumor volume was found in group D compared with that of group A, B and C (p < 0.05) from day 10 on. At day 20, mean tumor volumes of group D was significantly smaller than group A (*p* < 0.01), group B and C (*p* < 0.05). Tumor doubling time of group D (16.1 ± 0.7 d) was significantly prolonged in comparison with group A (7.8 ± 0.9 d), group B (7.3 ± 0.3 d) and C (8.0 ± 1.7 d) (*p* < 0.01). There was no significant difference (*p* > 0.05) of the tumor volume and tumor doubling time at the same time point between group A, B and C.

Spontaneous necrosis existed in A549 tumor with the necrosis ratio approximately 13% in each group as measured from CE-T1 MRI at baseline. After CA4P injection, a significantly increased necrosis ratio (*p* < 0.05) was obtained in group B and D. The necrosis ratio of group B and D stayed steadily till day 20. However, the necrosis ratio of group A and C increased steadily due to the tumor spontaneous necrosis occurrence during the tumor growing.

### Anatomical autoradiography

**Figure 5:**
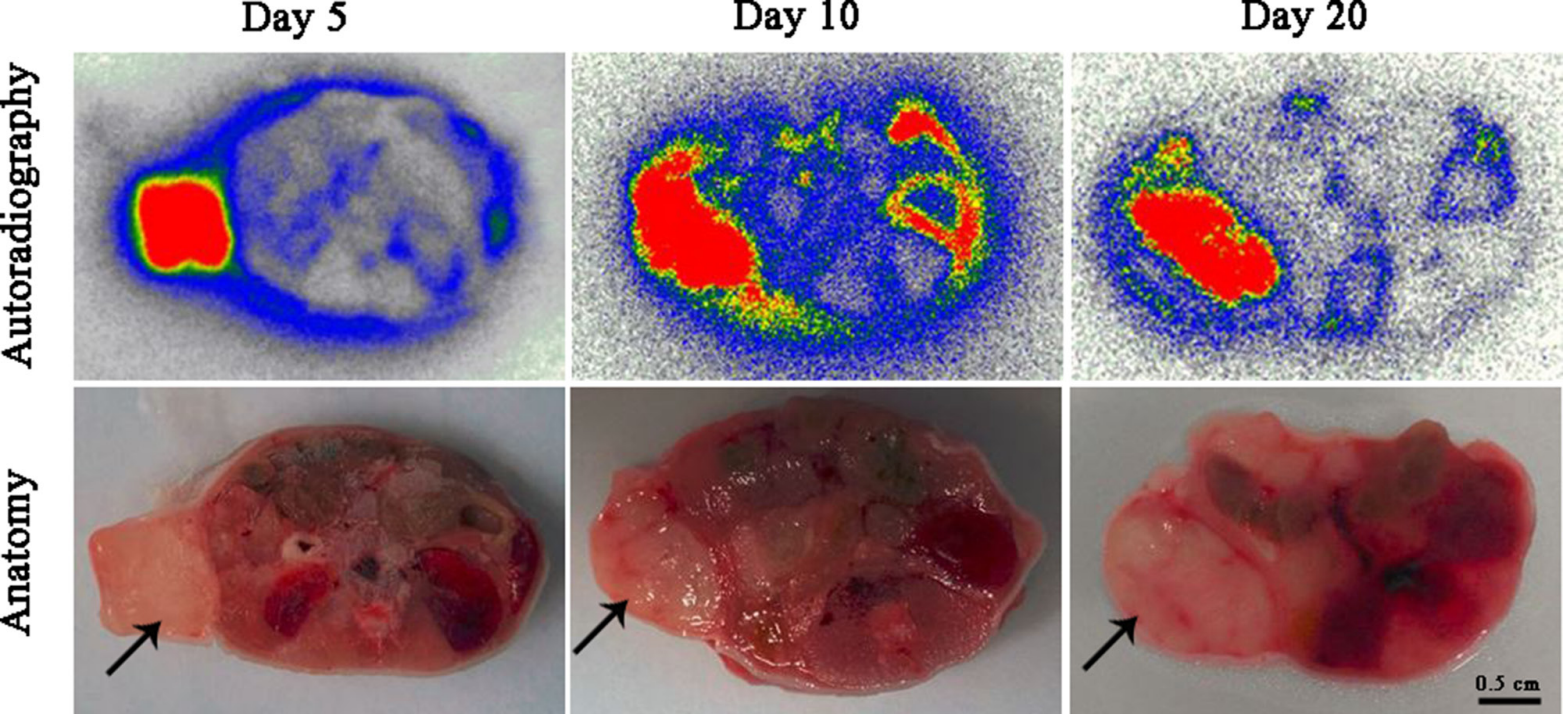
Anatomical autoradiography images of group D at day 5, 10, 20 after treatment Solid black arrow pointed to the tumor region. Autoradiography images (the up line), macroscopic anatomical photographs (the bottom line). High tracer uptake appeared in tumor region at each time point. Scale bar = 0.5 cm.

Anatomical autoradiography images of group D at day 5, 10, 20 after treatment are displayed in Figure 5. High tracer uptake appeared in tumor at each time point, which may facilitate imaging detection of tumor. The area of tracer uptake was approximate to the area of entire tumor at day 5 and 10. However, the tracer location appeared less than the area of entire tumor at day 20, which may be due to the regrows of tumor and the tracer located in the necrotic region of tumor.

### Toxicity analysis

Toxicity analysis (including blood indexes, hepatic and renal function indexes) in all groups of tumor-bearing nude mice with different drug treatment was carried out on day 20 (Table 2). The white blood cell numbers of group A were significantly lower than group B (*p* < 0.05), group C and D (*p* < 0.01). The higher white blood cell level of drug treatment groups might be caused by the therapeutic response of drugs. Other blood indexes in each group showed no significant difference (*p* > 0.05). These results revealed that ^131^I-prohy alone or combined therapy with CA4P displayed no obvious inhibitory effect on blood system and immune system.

**Table 2:** Toxicity analysis (including blood indexes, hepatic and renal function indexes) in all groups of tumor-bearing nude mice with different drug treatment at day 20

Parameter	Group A	Group B	Group C	Group D
WBC (10^9^/L)	3.89 ± 0.17	5.75 ± 0.69*	9.62 ± 0.21**	10.28 ± 0.01**
RBC (10^12^/L)	8.23 ± 2.04	9.88 ± 0.68	8.54 ± 0.99	7.77 ± 1.24
PLT (10^9^/L)	1113 ± 260	982 ± 133	1003 ± 159	1129 ± 91
HGB (g/L)	153 ± 45	181 ± 14	165 ± 23	152 ± 25
ALT (U/L)	32 ± 5	46 ± 3	41 ± 1	48 ± 6
AST (U/L)	76 ± 14	67 ± 4	92 ± 4	106 ± 11
ALB (g/L)	32 ± 8	41 ± 3	37 ± 1	32 ± 7
BUN (mmol/L)	6.79 ± 0.75	9.03 ± 0.17*	8.59 ± 1.70	8.80 ± 0.77
CREA (μmol/L)	13 ± 3	14 ± 2	16 ± 3	15 ± 2
UA (μmol/L)	155 ± 28	150 ± 12	113 ± 13	144 ± 18

WBC, white blood cell; RBC, red blood cell; PLT, platelet; HGB, hemoglobin; ALT, alanine aminotransferase; AST, aspartate aminotransferase; ALB, albumin; BUN, blood urea nitrogen; CREA, creatinine; UA, uric acid.

* *P* < 0.05

** *P* < 0.01 (comparison between group A and other groups).

Function indexes of liver (ALT, AST, ALB) and kidney (BUN, CREA, UA) were measured to evaluate the hepatic and renal damage after drug treatment. Hepatic and renal function indexes of group C and D revealed no obvious difference with group A (*p* > 0.05), which suggested no signs of tissue damage after treatment with ^131^I-prohy alone or the therapy combined with CA4P.

## DISCUSSION

In the present study, the biodistribution data from gamma counting and autoradiography revealed that ^131^I-prohy can be delivered efficiently to tumors. In addition, ^131^I-prohy combined with tumor vascular disrupting agent CA4P enhanced the uptake of ^131^I-prohy in necrotic tumor and prolonged survival of A549 tumor bearing nude mice compared with using ^131^I-prohy alone. From these prominent results, it can be concluded that TNTR appeared to be effective for the treatment of NSCLC.

The immune system functioned as a primary defense against cancer [13]. BALB/c nu/nu nude mouse lacks a thymus and is unable to produce T cells, which is therefore immunodeficient. For this reason, the anticancer efficacy could be mainly attributed to the drug effects rather than the natural immunity. The data from autoradiography and intratumoral biodistribution of drug fluorescence are consistent with gamma counting results, which revealed the tumor necrotic affinity of prohy. This property of prohy can be used for radionuclide imaging and TNTR. ^131^I-prohy used alone or in combination with CA4P showed some similar properties, such as high uptake in blood, heart, muscle and bone at early time points after administration and cleared rapidly afterwards. Both groups showed higher accumulation of ^131^I-prohy in mononuclear phagocyte system (lung, spleen and liver), necrotic tumor and viable tumor than other normal tissues. However, the radioactivity accumulation of CA4P + ^131^I-prohy group in necrotic tumor was significantly higher than that of ^131^I-prohy group at each detected time point (*p* < 0.05). CA4P induced tumor vascular shutdown and subsequent intratumoral necrosis, which provided extensive necrosis targets. This may be the reason why the radioactivity retention of CA4P + ^131^I-prohy group was higher than that of the group using ^131^I-prohy alone. The relevant results of TNTR in this study validated this suspicion.

In recent years, the anticancer effect is greatly improved by shifting from traditional cytotoxic chemotherapy to various targeted therapies [14]. Targeting to cancer-specific pathway, universally-vital targets, or tissues-specific targets showed different therapeutic advantages [15]. However, cell death is a universal feature existing in multiple pathological conditions, such as cardiovascular diseases and cancer [16]. Tumor spontaneous necrosis and drug induced tumor necrosis seem to be perfect tissues-specific targets with broad tumor spectrum and abundant targets for cancer treatment. In this study, ^131^I-prohy in combination with CA4P prolonged survival and inhibited tumor growth of nude mice in comparison to vehicle control group, CA4P control group and ^131^I-prohy control group. These results indicated an excellent anticancer effect of ^131^I-prohy to prevent rapid tumor growth and recurrence. Previous studies showed similar results by combining CA4P and ^131^I-labeled necrosis avid compound (hypericin and sennidin A) [17–19]. According to these promising results, TNTR may be a good approach for the treatment of solid tumors.

Monoclonal antibodies (mAbs) have been used as tumor targeting drug to directly inhibit tumor proliferation or to target drugs to tumors, and it has revolutionized tumor treatment and tumor imaging [20]. The mAb 14C5 has been developed for targeting tumor antigen 14C5, which is highly expressed in human cancer cell lines such as breast carcinoma (SK-BR-3, BT-20), lung carcinoma (A549), colon carcinoma (HT-29) and so on. Ingrid Burvenich et al. [21, 22] found that ^131^I-labeled mAb14C5 uptake peaked at 24 h in A549 NSCLC model and the activity accumulation in tumor was higher compared with that of ^131^I-prohy in combination with CA4P at the same time point in this study. However, almost all of normal organs and tissues showed higher radioactive retention of ^131^I-labeled mAb14C5 compared with ^131^I-prohy. The tumor-to-blood ratio of ^131^I-labeled mAb14C5 (about 1.0) was much lower than that of ^131^I-prohy in combination with CA4P (about 5.2). Less radioactive accumulation in normal tissues means less systemic toxicity that is beneficial for the clinical application of ^131^I-prohy. In addition, ^131^I-prohy as a low-molecular-weight radiopharmaceutical can avoid unwanted host immunological responses to foreign macromolecular mAbs, which often happens after treatment with mAbs [10, 23, 24].

TNTR provides a new approach for the treatment of NSCLC. In this study, ^131^I-prohy combined with CA4P did not show myelosuppression, hepatic and renal toxicity during tumor therapy, which are all crucial for propelling this anticancer strategy to clinical applications in human cancer patients [25]. This strategy can be applied to wide tumor spectrum and to treat primary and/or metastatic solid tumors basing on massive necrosis targets in the tumor provided by VDAs. Nevertheless, the exact mechanism for necrosis affinity of prohy remains unclear and the therapeutic effect need to be verified in other tumor models. Studies about these aspects will be made in the near future.

In conclusion, the current study demonstrated radionuclide can be delivered with prohy efficiently to tumors. Moreover, ^131^I-prohy combined with CA4P enhanced the uptake of ^131^I-prohy in necrotic tumor compared with ^131^I-prohy used alone. TNTR especially in combination with CA4P inhibited tumor growth, extended tumor doubling time and prolonged survival of A549 tumor bearing mice.

## MATERIALS AND METHODS

### Animals and tumor model

All animal care, use, and experimental procedures were approved by the Animal Affairs Committee of Jiangsu Academy of Traditional Chinese Medicine (Nanjing, Jiangsu, People's Republic of China). BALB/c nu/nu male nude mice (18–20 g), 4 to 6 weeks old, were provided by the Experimental Animal Center of the same agency. Human non-small cell lung cancer A549 cell line was obtained from KeyGen Biotech Co. Ltd (Nanjing, China), was cultured in RPMI-1640 supplemented with 10% fetal bovine serum (FBS, GIBCO), 100 units/ml penicillin, and 100 mg/ml of streptomycin at 37°C in 5% CO_2_. Xenografts were initiated by inoculating 1 × 10^6^ tumor cells subcutaneously into a unilateral flank region of mice. All animals were given Lugol's solution in drinking water to block thyroid gland from taking up free ^131^I.

### Drug preparations

Prohy was synthesized and purified using the method described by Falk et al [26]. The synthetic product was identified as prohy by HPLC-MS/MS, ^1^H-NMR, and ^13^C-NMR. The related parameters can be seen in our previous study [11]. The purity was over 98% as determined by HPLC. CA4P (purchased from HuaMei technology, Wuhan, China), was diluted in phosphate buffered saline (PBS) solution (10 mg/ml). Sodium iodide (^131^I, 740 MBq/mL) was provided by HTA (Beijing, People's Republic of China).

The Iodogen coating method was used for radiolabeling prohy. Briefly, prohy was dissolved in DMSO and mixed with Na^131^I solution (volume ratio, 4:1) and 50 μg Iodogen as the mixture solution. In order to decrease the percentage of DMSO used for injection, polyethylene glycol-400 and propylene glycol were used for dilution. The volume proportion of the mixture solution, polyethylene glycol-400 and propylene glycol was 1:7:7. The labeling yield was greater than 97% determined by HPLC with a pump (waters 2695, Millipore, Billerica, MA) and UV/Visible detector (waters 2998, Millipore, Billerica, MA) and HERM LB500 LC radio detector (Berthold Technologies, Bad Wildbad, Black Forest, Germany) and a Alltima C18 column (250 × 4.6 mm, 5 μm, Millipore, Billerica, MA). Mobile phase was methanol/ammonium acetate (6 mM) 85:15 at a flow rate of 1.0 mL/min. All experimental procedures were performed in the dark environment. The safety of oxidizing agent Iodogen has been proved by Miranda Cona et al [27]. Considering the high radiochemical purity of ^131^I-prohy (over 97%) and the low dosage of oxidizing agent (only 50 μg Iodogen) used in this study, ^131^I-prohy was not purified after preparation to remove Iodogen and the precursor prohy.

### Experimental protocols

**Figure 6:**
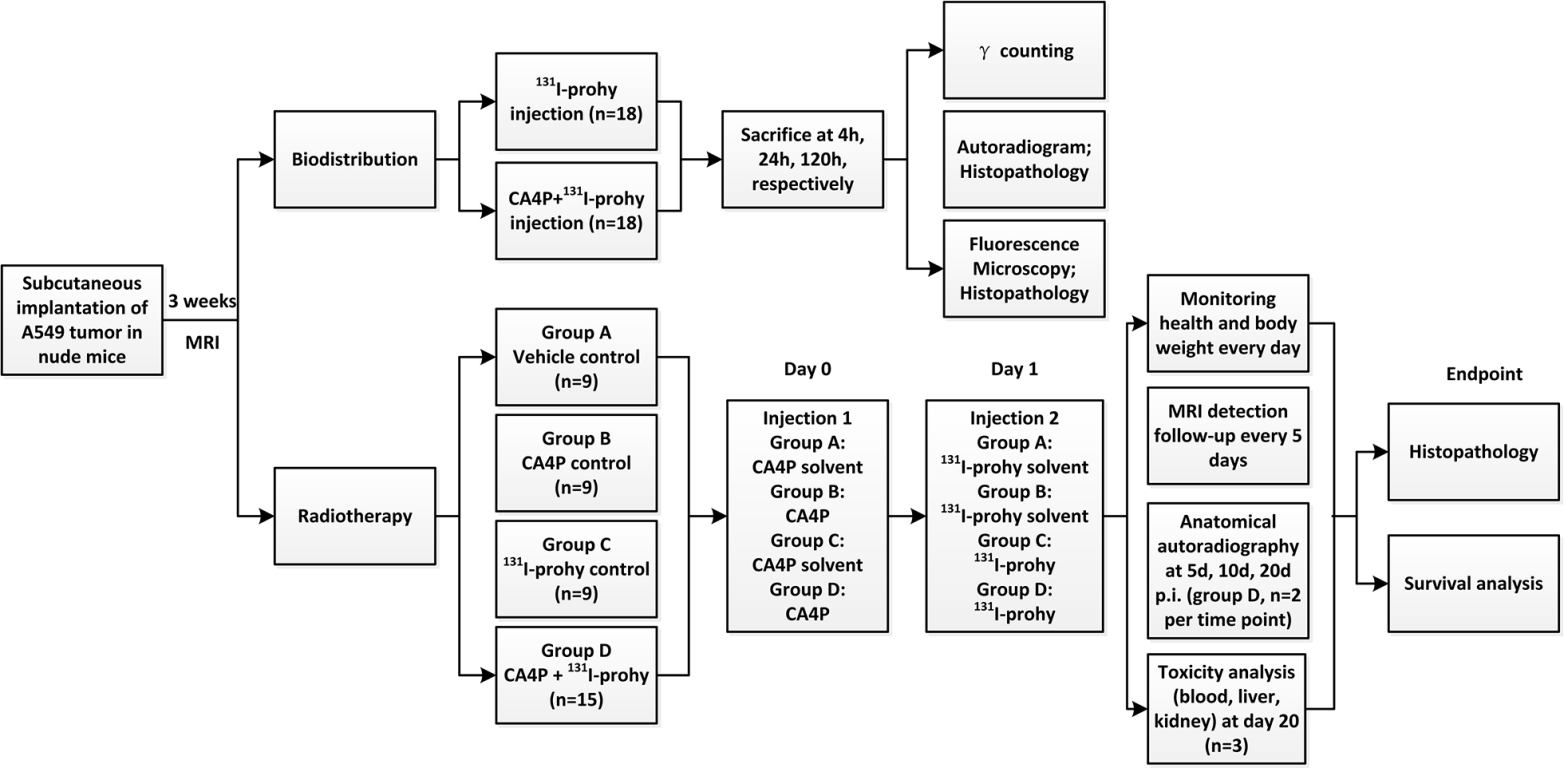
Flow diagram of experimental procedures in nude mice with unilateral subcutaneous A549 tumor xenografts (prohy: protohypericin)

Flow diagram of the whole experiment was displayed in Figure 6. Tumor size was assessed regularly with a vernier caliper using the formula width^2^ × length × 0.5. The experiment began when the tumor reached a diameter of 0.7 ± 0.2 cm three weeks after implantation. Nude mice were randomly divided into 2 major groups: one group was used for biodistribution analysis; another group was for TNTR study.

### Biodistribution analysis

Thirty six A549 tumor-bearing nude mice were divided into two subgroups. One group received *iv* injection of ^131^I-prohy, another group received sequential *iv* injections of CA4P (10 mg/kg) and ^131^I-prohy (18.5 MBq/Kg, 24 h after administration CA4P). During drug injection, animals were anesthetized using a small-animals gas anesthesia machine (Matrix VMP; GENE&I, Beijing, People's Republic of China) with isoflurane. Animals of each group were sacrificed by decapitation at 4 h, 24 h, 120 h (*n* = 6, each time point). Tissues of interest were harvested and washed with normal saline. After dried with gauzes, these tissues were weighted and measured the radioactivity with an automatic gamma counter (WIZARD; 2470, Perkin Elmer, New York, USA). Corrections were made for background radiation and physical decay during counting. All biodistribution results were expressed as percentage of the injected dose per gram of tissues (% ID/g).

### Autoradiography and histopathology

Tumor and liver at each time point of biodistribution analysis were sampled and cut into 50 μm frozen sections with a cryotome (Shandon, Thermo Fisher Scientific, Waltham, MA). Autoradiographs of these 50 μm Sections were obtained by 12–24 h exposure to a high performance storage phosphor screen. Images of the screen were read using Optiquant software (Cyclone; Canberra-Packard, Ontario, Canada). Subsequently, tumor sections were stained with haematoxylin-eosin (H&E) and digitally photographed. Relative tracer concentration in the necrotic tumor was estimated by manually drawing the regions of interest analysis for the necrotic and viable tumor as well as liver tissue on all autoradiographs. Necrotic and viable tumor was distinguished according to the histological examination of H&E stained sections. Autoradiograph of liver was used as a reference of radioactivity.

### Intratumoral location of prohy

Tumor at each time point for biodistribution analysis of ^131^I-prohy group were cut into 5 μm frozen sections and examined using a fluorescence microscopy (Vert A1, ZEISS, Gottingen, Germany) to acquire fluorescence images as prohy can emit red fluorescence. Fluorescence microscopy parameters: Rhod, filter set 20, excitation BP 546/12, beam splitter FT 560, emission BP 575–640. Afterwards, these sections were stained with H&E. The relative fluorescence density of the necrotic region and the normal region of tumor, which can be distinguished based on the histological examination of H&E stain, was calculated with ZEN software (Vert A1, ZEISS, Gottingen, Germany) by drawing the regions of interest.

### Tumor necrosis targeted radiotherapy study

Forty two tumor-bearing nude mice were divided into four subgroups (*n* = 9, each group, 6 extra animals were added in Group D for general phosphor screen analysis): group A of vehicle controls received *iv* injections of the solvents of CA4P and ^131^I-prohy; group B of CA4P control received CA4P (10 mg/kg) and solvent of ^131^I-prohy; group C of ^131^I-prohy control received ^131^I-prohy (185 MBq/Kg) and solvent of CA4P; group D of CA4P + ^131^I-prohy treatment received *iv* injections of CA4P (10 mg/kg) and ^131^I-prohy (185 MBq/Kg).

Body weight was recorded daily to monitor the health of nude mice. Animals of group D were sacrificed by overdose of anesthetic at day 5, 10, 20 post administration (*n* = 2 per time point) and cut into 0.5 cm slices after freezing for anatomical autoradiography analysis. Anatomical autoradiographs were obtained by 6 h exposure to a high performance storage phosphor screen to observe the biodistribution in the body of nude mice. Blood was collected at day 20 (*n* = 3 per group) for hematological biochemical parameters analysis including blood indexes, hepatic and renal function indexes (Modular DP, Roche Company, Mannheim, Germany) to evaluate the drug toxicity. Time to endpoint of every nude mouse was recorded for survival analysis. At endpoint, tumors were excised and cut into 5 μm frozen sections for histopathological findings. Histopathology of group A, B, C and D were carried out for postmortem verification.

### Magnetic resonance imaging (MRI)

MRI was performed before administration and every 5 days after administration to monitor and quantify tumor volume and necrosis using a clinical 1.5T MR magnet (Echo speed; GE, Milwaukee, WI). Nude mouse was anesthetized by a small-animals gas anesthesia machine (Matrix VMP; GENE&I, Beijing, People's Republic of China) with isoflurane and placed in a mouse holder in supine position for imaging acquisition. T1-weighted, T2-weighted and contrast enhanced T1-weighted (CE-T1) spin-echo multi-slice transverse images were acquired. Related parameters are transverse plane in all mice with slice thickness of 1 mm and a gap of 0 mm; T1WI (Sequences SE, TR/TE = 550 ms/24 ms); T2WI (Sequences FSE, TR/TE = 2920 ms/88 ms). CE-T1 (Sequences SE) was detected after *iv* injection of Magnevist (Bayer Schering Pharma AG, Berlin, Germany) at 0.2 mmol/Kg.

Tumor areas were manually delineated the outline of the tumor mass on CE-T1 MRI slices. Tumor volume was calculated use the following equation: tumor volume = Σ [tumor area on each slice × (slice thickness)]. Tumor necrotic area was delineated from CE-T1 images as central non-enhancing region to generate tumor necrotic volume. The ratios of necrosis were defined as the volume of necrosis over that of the entire tumor. Necrosis ratios were calculated based on the equation: necrosis ratio = Σ (area of necrosis × slice thickness) / (area of whole tumor × slice thickness) × 100%. Tumor doubling time (DT) was calculated according to the formula: DT = (T - T_0_) × log 2 / (log V – log V_0_), where (T - T_0_) represents the length of time between two measurements, V_0_ and V indicate the tumor volume at the two points of measurement [28].

### Survival analysis

For survival analysis, animal death was defined as the primary endpoint and was recorded every day. Standardized humane endpoint was used to euthanize animals, which referred to animals failed to eat and drink for over 3 days and without any limb movement.

### Statistical analysis

Statistical analysis was conducted with SPSS for Windows software package (version 17.0; Chicago, IL, USA). All quantitative data were expressed as mean ± SD. Two-tailed independent samples *t*-test was used for the comparison of biodistribution between ^131^I-prohy group and CA4P + ^131^I-prohy group. For survival analysis, Kaplan-Meier survival curves were made with *p* value generated from log-rank test. For other comparisons among groups, a one-way ANOVA was used. *P* value less than 0.05 was considered to be significant difference.

## Abbreviations

NSCLC: non-small cell lung cancer
TNTR: tumor necrosis targeted radiotherapy
TNT: tumor necrosis treatment
prohy: protohypericin
CA4P: combretastatin A4 Phosphate
VDA: vascular disrupting agent
H&E: haematoxylin-eosin
MRI: Magnetic resonance imaging
CE-T1: contrast enhanced T1-weighted
DT: tumor doubling time
mAbs: monoclonal antibodies
